# Antibiotic overuse, poor antimicrobial stewardship, and low specificity of syndromic case management in a cross section of men with urethral discharge syndrome in Kampala, Uganda

**DOI:** 10.1371/journal.pone.0290574

**Published:** 2024-03-15

**Authors:** Matthew M. Hamill, Annet Onzia, Rosalind M. Parkes-Ratanshi, Peter Kyambadde, Emmanuel Mande, Vivian Nakate, Johan H. Melendez, Ethan Gough, Yukari C. Manabe

**Affiliations:** 1 Division of infectious Diseases, Johns Hopkins School of Medicine, Baltimore, MD, United States of America; 2 Infectious Disease Institute, Kampala, Uganda; 3 Ministry of Health, National Sexually Transmitted Infections Control Program, Kampala, Uganda; 4 Johns Hopkins Bloomberg School of Public Health, Baltimore, MD, United States of America; The University of Sydney, AUSTRALIA

## Abstract

**Objective:**

High prevalence of sexually transmitted infections (STIs) combined with poor antimicrobial stewardship are drivers of STI antimicrobial resistance (AMR) especially in resource-limited settings where syndromic case management (SCM) is the norm. We characterized patterns of antibiotic use prior to clinic attendance and study enrollment in Ugandan men with urethral discharge syndrome (UDS), evaluated in-clinic prescribing, and the performance characteristics of SCM.

**Methods:**

Participants were recruited from government clinics participating in an existing gonococcal surveillance program in Kampala, Uganda. Questionnaires including antimicrobial use prior to attendance, prior episodes of UDS, penile swabs, and blood samples were collected. Bivariable and multivariable logistic regression models were used to estimate odds ratios (OR) for preselected factors likely to be associated with antibiotic use. In-clinic antibiotic treatment data were extracted from clinical notes, and the performance of SCM against laboratory-based STI diagnoses was evaluated.

**Findings:**

Between October 2019 and November 2020, 100(40%) of 250 men with UDS reported taking antibiotics in the 14days prior to attending the clinic. Of these 210(84%) had at least one curable STI and 20% had a reactive point-of-care HIV test. Multivariable analysis demonstrated significant associations between recent antimicrobial use and duration of UDS symptoms <6 days (OR 2.98(95%CI 1.07,8.36), p = 0.038), and sex with women only (OR 0.08(95%CI 0.01,0.82),p = 0.038). The sensitivity of SCM ranged from 80.0% to 94.4%; specificity was low between 5.6% and 33.1%. The positive predictive value of SCM ranged from 2.4(95%CI 0.7,6.0) for trichomoniasis to 63.4(95%CI 56.5,69.9) for gonorrhea.

**Conclusion:**

Pre-enrollment antibiotic use was common in this population at high risk of STI and HIV. Combined with the poor specificity of SCM for male UDS, extensive antibiotic use is a likely driver of STI-AMR in Ugandan men. Interventions to improve antimicrobial stewardship and deliver affordable diagnostics to augment SCM and decrease overtreatment of STI syndromes are required.

## Introduction

The dual epidemics of antimicrobial resistance (AMR) and sexually transmitted infections (STIs) facilitate the emergence and global spread of AMR STIs. The Centers for Disease Control and Prevention (CDC) categorizes the former as, “one of the biggest public health challenges of our time” [1]. In Europe and the United States, the estimated annual cost of AMR exceeded nine billion euros and 20 billion dollars, in direct costs, in 2013 and 2015 respectively [2]. In Africa, robust AMR data are lacking; in a 2017 review there were no AMR data from over 40% of countries in the continent [3].

Between 2012 [4] and 2016 [5] estimates of chlamydia, gonorrhea, syphilis and trichomoniasis increased globally from 357 to 376 million cases annually [5]. The Africa region, and Sub-Saharan Africa specifically has persistently high rates of curable STIs [6], though estimates are contested [7], and the highest prevalence of HIV globally [5, 8]. The consequences of sub optimally managed STIs can be severe and life-threatening [9]; STIs enhance transmission and acquisition of HIV [10, 11]. In resource limited setting (RLS), such as Uganda STI diagnostic infrastructure is lacking therefore syndromic case management (SCM) is used for empiric treatment of STI syndromes. However, SCM lacks specificity [12], can result in undertreatment, and unnecessary treatment [13, 14], resulting in increased risk for AMR. Evaluation of the performance of SCM in urethral discharge syndrome (UDS) in Africa are lacking, these analyses provide contemporary data in Ugandan men.

Previous work by our group has described the prevalence of HIV and curable STI in men in Kampala with UDS as well as patterns of STI AMR [15–19]. Here, we assessed antibiotic use in the 14 days prior to clinic attendance (henceforth pre-enrollment) in men with UDS, who presented to clinics participating in an existing Enhanced Gonococcal Antimicrobial Surveillance Program (EGASP) in Kampala, Uganda [17]. Additionally, we described the source and type of antibiotics used. Patterns of antibiotic treatments given or prescriptions provided by clinical staff (henceforth in-clinic) were identified, and the performance characteristic of UDS SCM were described using sensitivity, specificity, and positive predictive value (PPV) comparing SCM against gold-standard laboratory-based etiological diagnosis.

## Methods

Between October 2019 and November 2020 men with UDS, were recruited into a cross sectional study after completion of their usual care visit at government health centers, participating as EGASP sites in Kampala, Uganda. All participants were diagnosed and empirically treated by the clinical team using national UDS SCM guidelines prior to joining the study [20]; clinical care was not provided by the study team. UDS was defined as urethral discharge with or without dysuria. Self-reported UDS episodes in the 1-month and 6-months prior to study entry were recorded. Self-reported pre-enrollment antibiotic use data in the 14 days prior to joining the study were collected using structured questionnaires by trained research nurses. Demographic, and behavioral data, and self-collected genital swabs, and blood samples were collected as previously described [15]. Participants received Ugandan shilling 20,000 (equivalent to approximately $5–6) as transport reimbursement.

One of the authors (AO) had access to information that could identify individual participants during or after data collection; all data were deidentified prior to analysis. Each study participant was assigned a unique identification number that was used on study forms, in the study database, and on study specimens. None of the study forms, study database, or study specimens contained participant’s names or other information that could be used to identify them. The document linking subjects’ unique identification numbers with participants’ names and medical record numbers are kept in a locked office, and are not be accessible to personnel not associated with the study. All computers containing the study database are password- protected and the study database is not accessible by personnel not associated with the study.

### Sources of antibiotics

The antimicrobials described in the study were from two distinct sources. Pre-enrollment antibiotic use refers to the use of any antimicrobial agent reported by the participant in the 14 days *prior* to enrolling in the study. They were obtained from formal sources such as a doctor’s office or from a clinic after seeing a healthcare provider such as a medical officer or nurse; from a pharmacy where they could be purchased without a prescription, and from informal sources such as a store or from a street vendor. On attendance at the clinic, antimicrobials were also supplied to the patient by the clinic as treatment for UDS prior to study enrollment. These were standard-of-care antibiotics per Ugandan syndromic case management guidelines for UDS, and not provided by the study team. In-clinic treatments were either provided directly to the patient to be taken as directed or, if the medication was not available in the clinic due to stock outs, by prescription for the patient to present to an outside pharmacy whereupon he would pay for the medication(s). Ugandan guidelines for the treatment of UDS were cefixime 400mg once and doxycycline 100 mg twice daily for 7 days [20].

### Laboratory

The laboratory methods used have been previously described [15]. In brief, in Kampala, on-site point-of-care-tests (POCTs) were performed according to the manufacturer’s instructions for treponemal antibody (Laborex RDT, Zheijing Orient Gene Biotech Co. Ltd, China), HIV testing followed the rapid testing algorithm using the Determine (Alere, USA), Stat-Pak assay (Chembio Diagnostic Systems Inc., USA), SD-Bioline (Abbott Laboratories, USA). Gonococcal culture and sensitivity testing were conducted at the Infectious Disease Institute (IDI) laboratory in Kampala. Serum and genital swab elutes were transported to the Johns Hopkins University (JHU) International STD laboratory, Baltimore, MD for reverse sequence syphilis serological testing (chemiluminescence assay (Diasorin, Italy), Rapid Plasma Reagin (RPR) (Becton Dickinson, USA), and *Treponema pallidum* Particle Agglutination (TPPA) (Serodia, Japan)), and *Chlamydia trachomatis* (CT), *Neisseria gonorrhoeae* (NG), *Trichomonas vaginalis* (TV), and *Mycoplasma genitalium* (MG) testing by Aptima (Hologic Inc., USA) nucleic acid amplification tests (NAATs).

### Statistical analyses

Demographics, sexual and risk behaviors, and previous history of UDS were described for the total study sample and by reported pre-enrollment antimicrobial use using frequencies and proportions, or medians and interquartile ranges for categorical or numeric data, respectively. Differences between study participants by pre-enrollment antimicrobial use were determined by Fisher Exact test or Kruskal-Wallis test as appropriate.

We used bivariable logistic regression to determine demographic, behavioral or risk characteristics that explained the odds of pre-enrollment antimicrobial use. We fit a multivariable logistic regression model to identify participant characteristics that were independently associated with the odds of pre-enrollment antimicrobial use. Covariates were included based on literature review [21–24] and clinically relevant consideration including the following: age (< and ≥ 25 years), engagement in transactional sex, HIV status, presence of any curable STI, number of partners in the past 6 months, duration of UDS symptoms and sex of sex partners. Bias reduction was applied to logistic regression models to address data separation [25]. To compare the performance of SCM to laboratory-based etiological diagnosis of STI we calculated the sensitivity, specificity, and positive and negative predictive values of SCM. All analyses were performed in R version 4.2.0 software.

### Ethical oversight

All research was fully compliant with Good Clinical Practice principles. In Uganda, the Joint Clinical Research Center (protocol reference number JC0919), and the Ugandan National Council for Science and Technology (study number HS455ES) approved the study. The Johns Hopkins Institutional Review Board (IRB00215298) also reviewed the study. No study procedures were conducted until written informed consent had been obtained.

## Results

### Study population, STI prevalence

Men with UDS (n = 250) were recruited at 6 clinics in Kampala, Uganda. The study population and STI results have been previously presented [15]. Briefly, the proportion (95%CI) of participants with positive NAATs for NG, CT, TV, and MG were 66.4%(60.1%,72.2%) (n = 164), 21.7%(16.8%,27.4%) (n = 54), 2.0%(0.7%,4.9%) (n = 5), and 12.4%(8.1%,16.5%) (n = 31), respectively [15]. As previously reported, all available NG isolates (n = 142) were resistant to ciprofloxacin, penicillin, and tetracycline, but susceptible to extended spectrum cephalosporins (ESCs) and azithromycin [15]. HIV prevalence by sequential POCT was 20.0%(50/250), and 10.0%(25/250) had a reactive *T pallidum* antibody POCT.

Table 1 describes the demographic and behavioral characteristics and history of UDS of the overall study population. A quarter of participants were aged <25 years old, 95% reported sex with women only. A significant proportion of the participants reported high risk sexual behaviors including 148 (60.4%) of 247 condomless sex with partners of different or unknown HIV status, 101 (44.1%) alcohol use before sex, and 151 (61.1%) engagement in transactional sex. Table 1 also compared those who reported pre-enrollment antibiotic use with those who did not. Men who had sex with women only were less likely to report antibiotic use, people with HIV (PWH) were significantly more likely to have taken pre-enrollment antibiotics as were those with a previous history of UDS, and those with symptom duration of 6 days or more.

**Table 1:** Sociodemographics, clinical features and risk behaviors in men with urethral discharge syndrome in Kampala, Uganda.

	All	Reported pre-enrollment antimicrobial use^¥^	No reported pre-enrollment antimicrobial use^¥^	p-value
	N = 247^			
		N = 100	N = 147	
**Age**	24.00 [22.00,32.00]	24.00 [22.00,34.00]	24.00 [22.00,32.00]	0.537
Age: <25	129 (26.1%)	52 (26.0%)	77 (26.2%)	1.000
Age ≤24	129 (26.1%)	52 (26.0%)	77 (26.2%)	1.000
Age 25–35	76 (15.4%)	27 (13.5%)	49 (16.7%)	0.503
Age ≥36	42 (8.5%)	21 (10.5%)	21 (7.1%)	0.310
**Sexual behaviors**				
Sex with women only	235 (95.1%)	91 (91.0%)	144 (98.0%)	**0.016**
Sex with women and/or men	12 (4.9%)	9 (9.0%)	3 (2.0%)	**0.016**
Condom use sometimes/always	149 (60.3%)	56 (56.0%)	93 (63.3%)	0.290
No condom use in the last 6 months with partner of different/unknown HIV status	148 (60.4%)	65 (65.7%)	83 (56.8%)	0.185
Transactional sex	151 (61.1%)	63 (63.0%)	88 (59.9%)	0.690
Condomless sexual activity since UDS symptom onset	52 (21.2%)	29 (29.3%)	23 (15.8%)	**0.010**
Partner informed of symptoms yes	103 (41.7%)	53 (53.0%)	50 (34.0%)	**0.004**
Alcohol use last 6 M yes	119 (48.2%)	47 (47.0%)	72 (49.0%)	0.796
6 or more drinks on one occasion: Weekly	7 (2.9%)	5 (5.3%)	2 (1.4%)	0.124
6 or more drinks on one occasion: Daily or almost daily	2 (0.8%)	0 (0.0%)	2 (1.4%)	0.521
Alcohol use before sex past 6 M	101 (44.1%)	37 (41.1%)	64 (46.0%)	0.497
Positive HIV POCT	49* (19.8%)	29 (29.0%)	20 (13.6%)	**0.003**
Previous treatment for urethral discharge in past 6 M	107 (43.3%)	95 (95.0%)	12 (8.2%)	**<0.001**
Treated for UDS in past 1 month**	84 (78.5%)	83 (87.4%)	1 (8.3%)	**<0.001**
Number episodes of UDS in past 6 M	1.00 [1.00,2.00]	2.00 [1.00,2.00]	1.00 [1.00,2.00]	**<0.001**
Any curable STI positive	208 (84.2%)	85 (85.0%)	123 (83.7%)	0.860
Number partners past 6 M	2.0 [1.0,3.0]	2.0 [1.0,3.0]	2.0 [1.0,3.0]	**0.034**
		(range 1–60)	(range 1–20)	
Symptom duration ≥6 days	145 (58.7%)	78 (78.0%)	67 (45.6%)	**<0.001**

^¥^Reported use of antibiotics in the 14 days prior to study entry

^ Three participants had unknown antibiotic use

* This does not equal 50, 1 participant LWH had no antimicrobial use prior to clinic visit recorded.

** This is out of the 107 in the previous row.

*** total may exceed 100% as multiple responses could be given.

Agreement between point-of-care and laboratory tests for syphilis (N = 236) was high (area under ROC curve 0.94); the sensitivity and specificity of the POC tests were 88.5% [95CI 69.6,96.3] and 99.1% [95CI 96.3,99.8] respectively. Positive- and negative-predictive values were 92.0 [95%CI 72.9;98.0] and 98.6 [95%CI 95.7;99.5] respectively. The three false negative POCTs had negative RPRs in laboratory-based testing (S1 Table).

### Antibiotic use

#### Pre-enrollment antibiotic use in men with UDS in Kampala, Uganda

Table 2 describes those who reported antibiotic use in the 14 days prior to their study visit, including the type and source of antibiotic used, and duration of use. Antibiotic use was common; 100 (40.5%) reported use, the median duration of use was 5.0 days. Most, 89/100 (89%), had obtained antibiotics from a doctor, clinic, or pharmacy; 11% from non-healthcare settings such as store or on the street. No participants reported sourcing antibiotics from an on-line source or another source not listed. A wide range of antibiotics were reported including ciprofloxacin (26%), doxycycline (31%), and penicillin (16%). Others, such as trimethoprim-sulfur, were rarely reported (1%); 11% had used extended-spectrum cephalosporins and 4% azithromycin.

**Table 2:** Duration of use, source, and specific antimicrobial medication obtained in the 14 days prior to study enrollment visit.

	Reported pre-enrollment antimicrobial use N = 100
**Duration of antibiotic use** (days)	5.0 [3.0,6.5]
**Source of antibiotics**	
Bought on internet	0 (0.0%)
Bought on street	2 (2.0%)
Clinic	65 (65.0%)
Doctor’s office	7 (7.0%)
Family or friends	0 (0.0%)
Pharmacy	20 (20.0%)
Store	8 (8.0%)
**Type of antibiotic sourced**	
Azithromycin	4 (4.0%)
Cefixime	7 (7.0%)
Ceftriaxone	4 (4.0%)
Ciprofloxacin	26 (26.0%)
Doxycycline	31 (31.0%)
Metronidazole	14 (14.0%)
Other	7 (7.0%)
Penicillin	16 (16.0%)
Trimethoprim-Sulphur	1 (1.0%)

Of 164 participants with a positive NG NAAT 27/164 (16.5%) had a negative culture, of these 6/27 (22.2%) had a positive Gram stain. There was no statistically significant difference in concordance between NAAT and culture based on pre-enrollment antibiotic use P = 0.50.

Table 3 describes the bivariable and multiple logistic regression analysis of the association between participant characteristics and the pre-enrollment use of any antibiotic. A previous history of UDS treatment in the previous 6 months, and duration of symptoms for less than six days were positively associated with antibiotic use; sex with women only was negatively associated with pre-enrollment antibiotic use. None of the other associations that were significant in the bivariable analysis were robust to additional regression analysis.

**Table 3:** Bivariable and multiple logistic regression models of the association between patient characteristics and pre-enrollment antimicrobial use.

	Bivariable logistic regression OR (95%CI)	P value	Multiple logistic regression OR (95%CI)	P value
Age	1.01(0.99,1.04)	0.336		
Age group: <25 vs. ≥ 25	0.98(0.59,1.64)	0.953	1.71(0.55,5.35)	0.357
Sex with women only: Yes vs. No	0.23(0.06,0.86)	**0.028**	0.08(0.01,0.82)	**0.038**
Sex with men or women: Yes vs. No	4.29(1.17,15.76)	**0.028**		
Condom use in the last 6 months: Sometimes/always vs. Never	0.74(0.44,1.24)	0.255		
Condom use in the last 6 months with partner of different/unknown HIV status: Yes vs. No	1.44(0.85,2.45)	0.173		
Transactional Sex: Yes vs. No	1.14(0.67,1.92)	0.627	0.93(0.65,1.33)	0.700
Sexually active since UDS symptoms onset: Yes with condom vs. No	2.68(0.73,9.79)	0.137		
Sexually active since UDS symptoms onset: Yes without condom vs. No	2.33(1.24,4.35)	**0.008**		
Partner informed of symptoms prior to clinic visit: Yes vs. No	2.17(1.29,3.66)	**0.003**		
Alcohol intake in last 6 months: Yes vs. No	0.92(0.55,1.54)	0.763		
Alcohol before sex in the past 6 M: Yes vs. No	0.82(0.48,1.40)	0.469		
Positive rapid HIV test: Yes vs. No	2.57(1.35,4.86)	**0.004**	0.79(0.21,2.92)	0.719
Previous UDS treatment in past 6 M: Yes vs. No	188.22(66.49,532.82)	**<0.001**	160.27(52.42,490.00)	**<0.001**
Number UDS episodes past 6 M	1.52(1.08,2.15)	**0.016**		
Positive for any curable STI: Yes vs. No	1.09(0.54,2.21)	0.801	1.78(0.46,6.86)	0.401
Number of sex partners in past 6 M	1.06(1.98,1.15)	0.093	0.96(0.87,1.06)	0.424
Duration of symptoms in days <6 vs. ≥6	4.16(2.35,7.37)	**<0.001**	2.98(1.07,8.36)	**0.038**

#### Prescribing practices of treating clinician at the time of enrollment clinic visit

As described previously, adherence to Ugandan UDS guidelines by the treating clinician was high; overall 84.0% received or were prescribed cefixime 400mg, and 93.2% doxycycline 100mg twice daily for 7 days [15]. There were no significant differences overall in the treatment provided to patients who reported pre-enrollment antibiotic use compared to those who did not, with the exception of doxycycline which was significantly less likely to be offered to those with previous antibiotic use. Overall, 168 and one of 247 participants were prescribed metronidazole and tinidazole in-clinic (68.0% and 0.40% respectively); this was not significantly different by pre-enrollment antibiotic use p = 0.333.

There were no significant differences by pre-enrollment antibiotic use status and provision of medication directly by the clinic to the patient or provision with a prescription to purchase the medication from a pharmacy (S2 Table). Participants with HIV compared to those without were significantly more likely to be given UDS treatment at the clinic 2.21(1.00,4.72), p = 0.044. Participants with NG compared to those without were less likely to be given medication directly by the clinic, but sent away with a prescription to obtain medication elsewhere 0.28(0.08, 0.76),p = 0.023.

#### Sensitivity, specificity, and positive predictive value (PPV) of syndromic management of UDS-associated infections

The sensitivity, specificity, and positive and negative predictive values of syndromic management of specific UDS-associated infections are described in Table 4 (S3 Table). Sensitivity of SCM was 94.4% for CT, and <83% for NG and TV. In contrast, specificity of SCM was generally very low, only TV had a specificity of >6%. The PPV was 63.4% for NG but <22% in other curable STIs.

**Table 4:** Sensitivity, specificity, and positive and negative predictive values of syndromic case management by individual curable STI*.

	Lab Pos & Treated (TP)	Lab Neg & Treated (FN)	Lab Pos & Not Treated (FN)	Lab Neg & Not Treated (TN)	Sensitivity	Specificity	PPV	NPV
					(95%CI)	(95%CI)	(95%CI)	(95%CI)
**NG**	135	78	29	5	82.3(75.6,87.8)	6.0(2.0,13.5)	63.4(56.5,69.9)	14.7(5.0,31.1)
**CT**	51	184	3	11	94.4(84.6,98.8)	5.6(2.8,9.9)	21.7(16.6,27.5)	78.6(49.2,95.3)
**TV**	4	164	1	18	80.0(28.4,99.5)	33.1(27.2,39.3)	2.4(0.7,6.0)	98.8(93.4,100.0)

* Unknown antibiotic use data in 3 participants

## Discussion

There are two key findings in our study of young men with UDS in Kampala. One was the very high community usage of antibiotics, four in ten men received treatment for UDS prior to their clinic visit. This implies that there might be a larger group of men who take treatment with resolution of symptoms but not cure who never seek medical care but who may still transmit STIs. The practice of antibiotic self-medication is common in Africa; in one Ugandan study, 76% of general population respondents acknowledged that antimicrobial self-medication was associated with poor outcomes, of these 33.2% cited drug resistance as a risk [26]. The second key finding was the overuse of antibiotics by healthcare workers driven, in part, by SCM. Overconsumption and overprovision of antibiotics amplifies the risk of AMR; AMR organisms are a threat to global health security [27]. In East Africa, bacteria with high levels of AMR to commonly-used antibiotics have been reported [28] as well as to antibiotics typically reserved for more serious infections [29]. Additionally, antibiotic provision by non-clinicians, particularly pharmacies [30, 31], in a non-regulated manner is likely to exacerbate AMR [32]. Studies have demonstrated unregulated provision of antibiotics by pharmacists in numerous low- and middle-income settings (LMIC) including Syria [30], Sri Lanka [33], Sub Saharan Africa [34, 35] including Nigeria [36] and Uganda [37]. An international review of 13 studies [38] described poor knowledge about AMR or provision of antimicrobials in pharmacies in ways likely to promote AMR. Additionally, legal prohibitions on the provision of antibiotics without prescription in LMICs are either lacking or poorly enforced [39]; some countries such as Thailand have developed a national strategy on AMR that strengthens legal sanctions [40]. Community engagement with key opinion leaders in local communities have demonstrated reductions in antibiotic overuse in pharmacies and village groceries in Thailand [41] and other LMICs [39].

The high prevalence of gonorrhea (66.4%), and MG (12%) positivity among men with UDS, coupled with high community-based antibiotic use increases concerns for ongoing AMR selection pressures for these two STIs specifically, as well as other bacteria of public health importance [42]. Previous work by our group in Uganda has described macrolide resistance in 10.7% of those with MG [16]. MG is unlikely to be eradicated by the doxycycline used in SCM; in a Swedish study only 38% of men achieved cure with doxycycline therapy [43]. The efficacy of doxycycline in the treatment of MG in Ugandan men with UDS is unknown.

Almost 30% of participants took ciprofloxacin, penicillin, or doxycycline pre-enrollment, this will not have eradicated NG but may have reduced diagnostic sensitivity, particularly by culture, by partially suppressing bacterial replication; this is suggested by the finding that over one fifth of culture-negative samples had a positive Gram stain. However, there are several other reasons for NG culture failure such as delays in transporting samples to the laboratory, and environmental conditions including high ambient heat and humidity typical in Kampala. The observed CT and TV positivity may be an underestimate because 14.0% and 5.6% reported pre-enrollment doxycycline/azithromycin and metronidazole exposure, respectively. The prevalence of TV was only 2.0% however almost 70% were treated on the day of clinic visit with 5-nitroimidazoles which may cause AMR in TV, and clinically important anaerobes [44]. Furthermore, pre-enrollment doxycycline/azithromycin/penicillin exposure may have blunted serological responses in those with syphilis, underestimating the proportion with high RPR titers; 56% of those treated for syphilis with a single 1g dose of azithromycin in Rakai, Uganda had seroreversion or a ≥4-fold decrease in RPR at 10 months [45].

Pre-enrollment antibiotic use was more common in those with shorter duration of symptoms possibly because these men had more severe symptoms so sought out antibiotic treatment early after symptom onset. Participants with a positive NG NAAT compared to those without were less likely to be given medication directly from the clinic, but sent away with a prescription to obtain medication elsewhere. This increases the risk of onward transmission, no treatment or incorrect treatment emphasizing the need for diagnostic certainty. Men with HIV were more likely to use antimicrobials pre-enrollment and to be provided with medication directly in-clinic, this observation requires further investigation but may reflect better health literacy in those with HIV who are linked with healthcare services.

SCM performance was poor in men with UDS in Kampala as has been noted in other settings [12, 46, 47]. Specificity of SCM was very low, further contributing to antibiotic overuse and resulting in inappropriate use of limited financial resources. A Gram stain of urethral discharge is a quick and sensitive POC test for gonococcal and non-gonococcal urethritis. However, a serviced microscope, reliable electricity, replacement bulbs, trained staff, proficiency testing, and reagents are scare in a LMIC like Uganda precluding widespread adoption. The availability of rapid diagnostics such as POC microscopy or POC NAAT could revolutionize UDS management by providing personalized care and dramatically improve antimicrobial stewardship [48, 49].

The strengths of this study include gold standard diagnosis of UDS-associated STIs in a CAP-certified laboratory. Clinic-based treatment data were collected from the medical record so likely to be reliable. The participants were adult males with UDS attending government health centers in Kampala, and data may be generalizable to these settings. The study is limited by its focus on symptomatic urethritis, modest sample size, and limited geographic coverage which precludes extrapolation to other populations in Uganda. Additionally, we did not collect data on why in-clinic antimicrobials were given either directly to the patient or via a prescription. We retained Previous Treatment for UDS in our multivariable regression model, despite a very large OR and confidence intervals, because removing the variable from our model is not recommended when it was important for us to adjust for its effects [50]. However, despite the use of a bias corrected model, due to the small number of participants in that group who did not report pre-clinic antibiotic use (N = 12) the association between these variables could not be estimated precisely. Self-reported antibiotic use data are beset with recall and social desirability biases, although the 14-day window should have minimized the former.

### Conclusion

Syndromic STI case management, poor antibiotic stewardship, and high prevalence of bacterial STIs represents a perfect storm for STI AMR selection in men with UDS in Kampala. Studies are needed to better understand knowledge, attitudes, and behaviors, and other drivers of antimicrobial consumption in men with UDS. Additionally, studies to assess antibiotic-provider knowledge of etiological causes of STI, Ugandan STI treatment guidelines, and principles of antimicrobial stewardship are required. Qualitative research to understand the drivers and facilitators of antibiotic overuse in this setting can support development of appropriate interventions for antibiotic users and providers alike. Ultimately, more community engagement and participation which was shown to reduce antibiotic use in Thailand; sweeping legal measures may be required or more strictly enforced in Uganda to restrict unregulated provision of antimicrobials and help prevent the expansion of AMR.

## Supporting information

S1 Table Sensitivity, specificity and negative and positive predictive values of point-of-care tests for syphilis antibodies. (DOCX)

S2 Table Prescribing practices of treating clinician at the time of enrollment clinic visit. (DOCX)

S3 Table In-clinic antimicrobial provision to participants attending government health centers in Kampala, Uganda who were positive or negative for each organism. (DOCX)
