# Supplementary material for: Antibiotic overuse, poor antimicrobial stewardship, and low specificity of syndromic case management in a cross section of men with urethral discharge syndrome in Kampala, Uganda
Source: PLoS One. 2024 Mar 15;19(3):e0290574. doi: 10.1371/journal.pone.0290574 (PMC10942085; doi:10.1371/journal.pone.0290574)
Supplement: S1 Table — (DOCX) [file pone.0290574.s001.docx]

**S1 Table. Sensitivity, specificity, and negative and positive predictive value of point-of-care tests for syphilis antibodies**

| **Lab-based test** | **POC test** | | | | | | |
| --- | --- | --- | --- | --- | --- | --- | --- |
|  | **Neg** | **Pos** | **Total** | **PPV**  **Neg** | **NPV**  **Pos** | **Total** |  |
| Neg | 208  (99.05[96.25;99.76])^^^ | 2  (0.95 [0.24;3.75]) | 210  (100) | 208  (98.58 [95.66;99.54])^α^ | 2  (8.00 [1.99;27.09]) | 210  (88.98) |  |
| Pos | 3  (11.54 [3.75;30.4]) | 23  (88.46 [69.58;96.25])^*^ | 26  (100) | 3  (1.42 [0.46;4.34]) | 23  (92.00 [72.91;98.01])^∞^ | 26  (11.02) |  |
| Total | 211  (89.41) | 25  (10.59) | 236  (100) | 211  (100) | 25  (100 | 236  (100) |  |

Frequency, Row %[95%CI]

^*^Sensitivity: 88.46%

^^^Specificity: 99.05%

^α^Positive predictive value (PPV): 92.00%

^∞^NPV negative predictive value (NPV): 98.58%

Laboratory-based Treponemal test at JHU in Baltimore. POC, POC treponemal antibody test used in Uganda
