# Supplementary material for: Antibiotic overuse, poor antimicrobial stewardship, and low specificity of syndromic case management in a cross section of men with urethral discharge syndrome in Kampala, Uganda
Source: PLoS One. 2024 Mar 15;19(3):e0290574. doi: 10.1371/journal.pone.0290574 (PMC10942085; doi:10.1371/journal.pone.0290574)
Supplement: S2 Table — (DOCX) [file pone.0290574.s002.docx]

**S2 Table. Prescribing practices of treating clinician at the time of enrollment clinic visit**

|  | **All** | **Reported pre-enrollment**  **antimicrobial use** | **No reported antimicrobial exposure prior to clinic visit** | **P-value** |
| --- | --- | --- | --- | --- |
| Cefixime 400mg yes | 210 (85.0%) | 82 (82.0%) | 128 (87.1%) | 0.282 |
| Ceftriaxone 500mg | 3 (1.2%) | 3 (3.0%) | 0 (0.0%) | 0.065 |
| Azithromycin 1 g | 2 (0.8%) | 1 (1.0%) | 1 (0.7%) | 1.000 |
| Azithromycin 2 g | 1 (0.4%) | 1 (1.0%) | 0 (0.0%) | 0.405 |
| Other treatment yes | 37 (15.0%) | 17 (17.0%) | 20 (13.6%) | 0.473 |
| Doxycycline/tetracycline | 230 (93.1%) | 88 (88.0%) | 142 (96.6%) | **0.011** |
| Approved UDS treatment [cefixime AND doxycycline] | 207 (83.8%) | 80 (80.0%) | 127 (86.4%) | 0.218 |
| Metronidazole OR Tinidazole | 169 (68.4%) | 72 (72.0%) | 97 (66.0%) | 0.333 |
| First of 2 medications (ESC) given directly to patient | 191 (78.0%) | 81 (81.0%) | 110 (75.9%) | 0.353 |
| patient given prescription for 1^st^ drug | 54 (22.0%) | 19 (19.0%) | 35 (24.1%) | 0.353 |
| Second of 2 medications (Doxycycline) given directly to patient | 213 (88.0%) | 85 (86.7%) | 128 (88.9%) | 0.688 |
| patient given prescription for 2^nd^ drug | 29 (12.0%) | 13 (13.3%) | 16 (11.1%) | 0.688 |

ESC, extended spectrum cephalosporins; mg, milligrams; g, grams
