# Supplementary material for: Antibiotic overuse, poor antimicrobial stewardship, and low specificity of syndromic case management in a cross section of men with urethral discharge syndrome in Kampala, Uganda
Source: PLoS One. 2024 Mar 15;19(3):e0290574. doi: 10.1371/journal.pone.0290574 (PMC10942085; doi:10.1371/journal.pone.0290574)
Supplement: S3 Table — (DOCX) [file pone.0290574.s003.docx]

**S3 Table. In-clinic antimicrobial provision to participants attending government health centers in Kampala, Uganda who were positive or negative for each organism.**

| **Organism** | **Antimicrobial treatment provided**  **n, (%[95%CI])** |
| --- | --- |
|  | **Extended Spectrum Cephalosporin** |
| NG Pos | 135 (82.3%[75.4,87.7]) |
| NG Neg | 78 (94.0%[85.9,97.8]) |
| NG Pos alone | 95 (81.9%[73.4,88.2]) |
|  |  |
|  | **Doxycycline OR Azithromycin** |
| CT Pos | 51 (94.4%[83.7,98.6]) |
| CT Neg | 184 (94.4%[89.9,97.0]) |
| CT Pos alone | 17 (94.4%[70.6,99.7]) |
|  |  |
|  | **Doxycycline OR Azithromycin** |
| MG Pos | 30 (96.8%[81.5,99.8]) |
| MG Neg | 206 (94.1%[89.8,96.7]) |
| MG Pos alone | 11 (91.7%[59.8,99.6]) |
|  |  |
|  | **Metronidazole OR Tinidazole** |
| TV Pos | 4 (80.0%[29.9,98.9]) |
| TV Neg | 164 (66.9%[60.6,72.7]) |
| TV Pos alone | 0/0 |

Pos, positive laboratory NAAT test for: *Neisseria gonorrhoeae* (NG), *Chlamydia trachomatis* (CT), *Mycoplasma genitalium* (MG), *Trichomonas vaginalis* (TV). Neg, negative
